# Magnetic resonance imaging and computed tomography as tools for the investigation of sperm whale (*Physeter macrocephalus*) teeth and eye

**DOI:** 10.1186/s13028-017-0307-y

**Published:** 2017-06-12

**Authors:** Aage Kristian Olsen Alstrup, Ole Lajord Munk, Trine Hammer Jensen, Lasse Fast Jensen, Abdi Hedayat, Brian Hansen

**Affiliations:** 1 Department of Nuclear Medicine and PET Center, Institute of Clinical Medicine, Aarhus University Hospital, Nørrebrogade 44, 10C, 8000 Aarhus, Denmark; 2 Department of Chemistry and Bioscience, Aalborg University/Aalborg Zoo, Mølleparkvej 63, 9000 Aalborg, Denmark; 3 Fisheries and Maritime Museum, Tarphagevej 2, 6710 Esbjerg, Denmark; 4 Natural History Museum of Denmark, University of Copenhagen, Universitetsparken 15, 2100 Copenhagen, Denmark; 5 Center of Functionally Integrative Neuroscience, Aarhus University Hospital, Nørrebrogade 44, 10G, 8000 Aarhus, Denmark

**Keywords:** Imaging techniques, Computer tomography, Magnetic resonance, Sperm whales, Eyes, Teeth

## Abstract

**Background:**

Scanning techniques such as magnetic resonance imaging (MRI) and computed tomography (CT) are useful tools in veterinary and human medicine. Here we demonstrate the usefulness of these techniques in the study of the anatomy of wild marine mammals as part of a necropsy. MRI and CT scans of sperm whale teeth (n = 4) were performed. The methods were compared and further compared to current standard methods for evaluation of tooth layering. For MRI a zero echo time sequence was used, as previously done for imaging of intact human teeth. For CT two different clinical scanners were used.

**Results:**

The three scanners did not provide sufficient information to allow age estimation, but both MRI and CT provided anatomical information about the tooth cortex and medulla without the need for sectioning the teeth. MRI scanning was also employed for visualizing the vascularization of an intact eye from one of the stranded sperm whale.

**Conclusions:**

Clearly, MRI was useful for investigation of the retinal vasculation, but optimum results would require well-preserved tissue. It was not possible to estimate age based on CT scans of tooth growth lines. Further research is needed to clarify the usability of MRI and CT as tools for marine mammal research when samples need to remain intact or when a spatial (three dimensional) arrangement of features needs to be determined.

## Background

Traditionally, anatomical and pathological knowledge in cetacean research is obtained by dissection of stranded individuals followed by e.g. bacteriological, virological, morphological or histological examinations [1–3]. However, modern scanning techniques have much to offer in this research field—at least as a supplement to dissection: first, modern scanners offer the possibility of non-invasive imaging of organs providing access to data about organ structure in situ, e.g. vasculature of soft tissue organs such as the eye, heart or kidney. Second, many museums find preservation of the intact organ desirable, which can be compromised by traditional examination techniques, in particular necropsy. In toothed whales, for instance, age is estimated by counting growth layers in the teeth after cutting them into halves, which makes the teeth less suited for later display. Non-invasive analyses are also preferable when dealing with samples originating from rare or extinct species. Third, modern imaging techniques and computer aided data visualization offer a better three-dimensional understanding of the organ morphology than simple dissection, which is a benefit e.g. for bone examination in whales [4]. Today, three-dimensional imaging techniques can provide anatomical information more quickly and in a non-invasive way, as described for terrestrial vertebrates by Lauridsen et al. [5].

Both magnetic resonance imaging (MRI) and computed tomography (CT) provide detailed images with millimeter spatial resolution for routine scans and cellular level resolution is attainable when imaging samples [6–9]. In MRI a constant external magnetic field sets up a magnetization in the subject or sample being imaged. This magnetization may be manipulated with radiofrequency pulses to induce a signal in a detector coil. During the scan the magnetization is encoded in frequency and phase by additional time-dependent magnetic fields (called gradients). This encoding makes it possible to reconstruct an image from the signal received in the coil. MRI offers very good soft tissue contrast, but MRI in bony structures is often not feasible due to the very rapid signal decay in these crystalline structures. In comparison, medical CT scanners consist of a rotating X-ray source, which is rigidly linked to an X-ray detector located on the other side of the target [10]. The target is exposed to a fan-shaped X-ray beam, while being translated through the scanner. The CT detectors measure the X-ray transmission through the target at all angles, which allows reconstruction of a three-dimensional density image. The resulting CT image is linearly rescaled into CT numbers in Hounsfield units.

CT is a fast and useful technique for visualizing the normal and pathological anatomy of organs, which has been particularly well-suited for imaging of mineral or crystalline structures such as bones and teeth. For clinical use, contrast-enhanced CT protocols with intravenously or orally administrated iodine-based contrast agents are routinely used to improve tissue differentiation and delineation of anatomic structures, and for ex vivo imaging, iodine staining of soft tissue specimens has recently been shown to improve visualization using diffusible iodine-based contrast-enhanced computed tomography (diceCT) [11]. Both MRI and CT are routinely used in human and veterinary medicine, and they are also useful tools in preclinical and clinical research [12–14].

Recent technological developments have improved both resolution and versatility of MRI and CT scanning techniques. For instance, using ultra-short echo time (UTE) sequences [15, 16] and more recently, zero echo time (ZTE) sequences [17–19] allow MRI to be used on bony structures as well. Thus, Weiger et al. [20] showed that the ZTE technique provides a good delineation of mineralized dentine and enamel layers in human teeth. Likewise, recent developments in CT scanning and reconstruction techniques have increased the resolution significantly.

In cetaceans, scanning techniques have proved useful in anatomical examinations and have been applied in studying e.g. head anatomy of Cuvier’s beaked whale (*Ziphius cavirostris)* [21–23] and sperm whale (*Physeter macrocephalus*) [24], brain anatomy of the white whale (*Delphinapterus leucas*) [25] and dwarf sperm whale (*Kogia simus*) [26] and in discovering a sensory organ related to lunge feeding in rorqual whales (*Balaenopteridae*), a technique used to engulf large quantities of concentrated prey [27]. However, scanning techniques are not applied on a routine basis in cetacean research due to the difficulties of getting access to fresh tissue or organ samples as well as logistical challenges and the sheer size of many cetacean species.

In this study the usage of modern MRI and CT scanning techniques in anatomical examinations of sperm whales was evaluated for two challenging samples: thick dense teeth with fine inner structures and an eye with detailed soft-tissue structures. Specifically, we investigate whether MRI and CT scanning of sperm whale teeth are suitable methods for age estimation by identifying tooth growth lines [28] and resolving details of anatomy, including measurement of dentin and enamel thickness. In addition, the anatomical details of retina vasculation in situ and sclera structure in the sperm whale eye were studied by MR imaging and compared to similar scans acquired in an isolated pig eye. This demonstrated possibilities and limitations of using modern clinical and preclinical imaging techniques in cetacean research, for which samples are often not in an ideal state due to various stages of decomposition.

## Methods

**Fig. 1:**
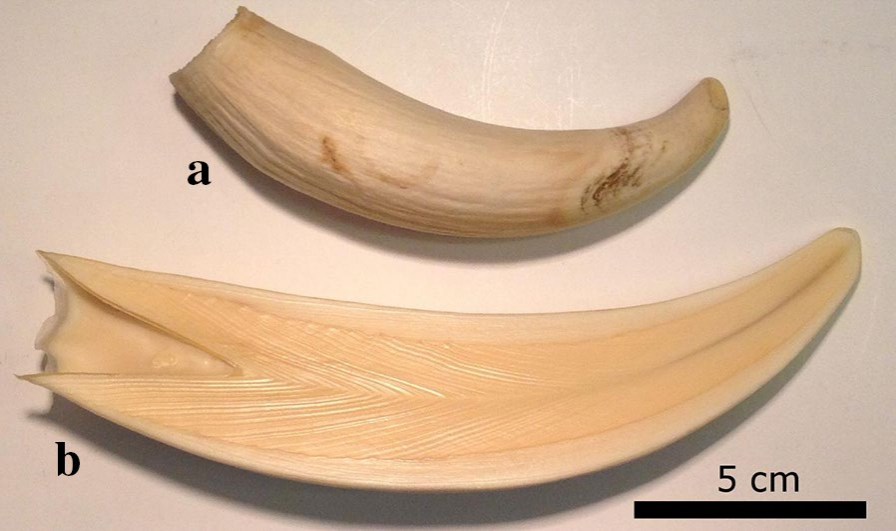
The two whale teeth (tooth **a** MCE1652 and tooth **b** MCE1654) used for evaluation of the use of ZTE MRI in whale age estimation

Teeth (Fig. 1) were sampled from a whale bone collection belonging to the Natural History Museum of Denmark, Copenhagen, Denmark. The teeth (n = 4) were from stranded male sperm whales stranded in 1996 (MCE1654; Rømø, Denmark), 1997 (MCE1652; Rømø, Denmark) and 2014 (MCE1644 and MCE1645; Henne Strand, Denmark). Due to the limited gantry size only teeth from MCE1652 (tooth A) and MCE1654 (tooth B) were MRI scanned, while all four teeth were CT scanned. In 2014, the eye was taken from a stranded whale (MCE1645) a few hours after death at Henne Strand, Denmark. The whale was still alive when it stranded, and the eye was immediately preserved in 4% buffered formaldehyde post mortem. After 2 weeks, the eye was transferred to a 99% ethanol solution until scanning was performed. Similarly, a pig eye was sampled from a female 40 kg domestic pig (*Sus scrofa domesticus*) after euthanasia as part of a different research experiment (approved by The Danish Animal Experiments Inspectorate). The pig eye was processed in the same way as the whale eye.

**Fig. 2:**
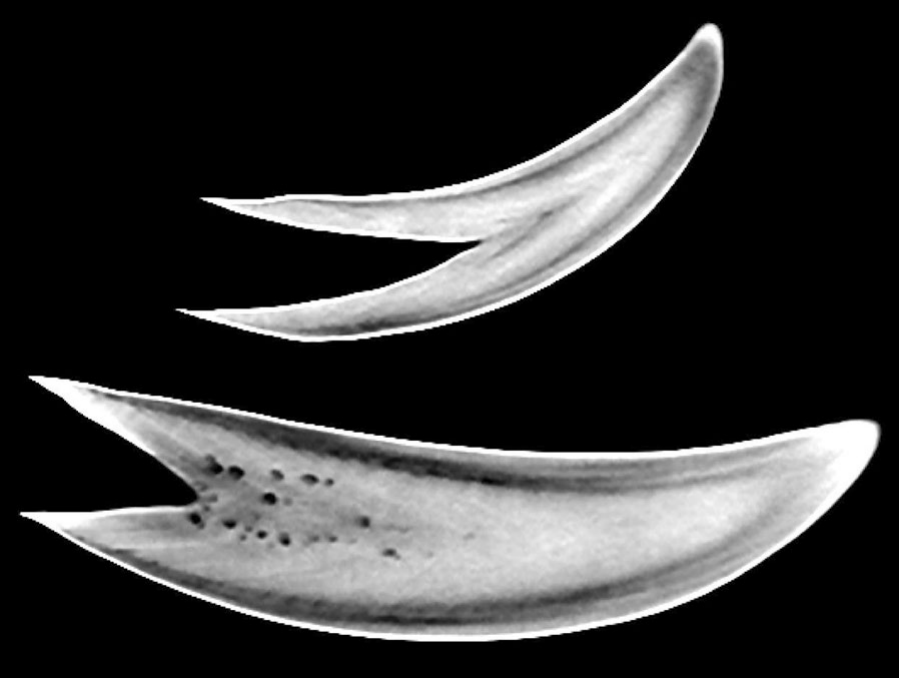
CT images of both teeth (tooth A and B) using Somatom Definition Flash and the scan- and reconstruction parameters in the CT method details. Coarse inner structures such as *small inner holes* and different densities of enamel and marrow are clearly visualized. Parts of the V-shaped structures can be seen

**Fig. 3:**
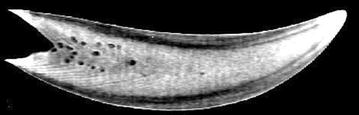
CT image of tooth B using Biograph 64 and the scan- and reconstruction parameters in the CT method details. The slice is similar (but not exactly the same) as in Fig. 2. This image has smaller slice thickness and the filter was not as smooth as in Fig. 2. Thus, this image has more noise but higher spatial resolution, which improves visualization of the fine V-shaped pattern. Still only parts of the V-shaped structures can be visualized and the total number of layers cannot be determined from these images

CT of the whale teeth was performed on two clinical CT scanners: Siemens Biograph 64 Truepoint PET/CT and Siemens Somatom Definition Flash. These or similar modern scanners are widely available. Images of the teeth were comparable for the two scanner systems (Figs. 2 and 3), but the scan settings and reconstruction parameters were important for the quality of the images and visualization of the inner structures of the teeth. We aimed at using parameters for high-resolution imaging. Details of the scan and reconstruction parameters are provided below. The eyes were not CT scanned because good soft tissue CT imaging requires the use of contrast agent, which is only possible for in vivo studies. Thus, MRI is more suitable for this purpose.

MRI of teeth and eyes was performed on a Bruker Biospec 9.4T animal system. Scan details are provided below. Anatomical scans of the eye were performed along three orthogonal directions to ensure a detailed mapping of the anatomical features. For mapping the vasculature of the eye, including the retina we used a fast gradient echo scan which to our experience is very sensitive to the vasculature in this type of sample where blood was not washed out prior to preservation. After scanning, vascular segments in each image plane were segmented out by hand and processed in Matlab (The Mathworks, Natick, MA, USA). In this manner, mapping of the eye vasculature was possible.

### MRI method details

Eye vasculature was imaged using an 86 mm quadrature coil and a gradient echo (FLASH) sequence: resolution 180 µm × 180 µm × 500 µm, echo time: 7.2 ms, repetition time: 2.3 s, 20 averages, scan time: 5 h.

The eye anatomy was imaged using a 3D MDEFT sequence. Resolution in the pig eye scans was 120 µm × 120 µm × 250 µm, echo time: 3.5 ms, repetition time: 4.2 s. In the whale eye the resolution was 466 µm × 466 µm × 700 µm.

**Fig. 4:**
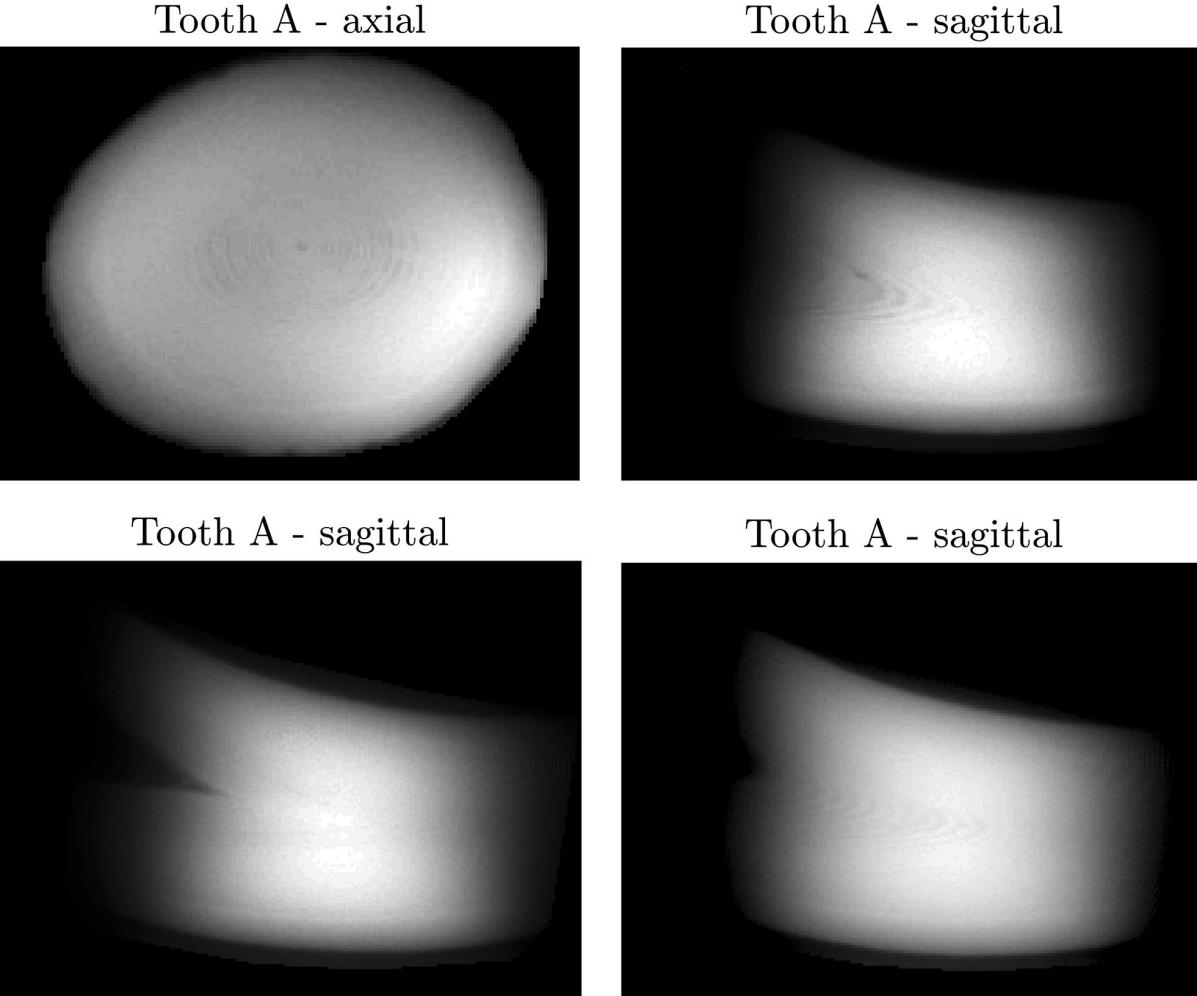
Examples of ZTE images in tooth *A* (see Fig. 1) along various axis (axial and sagittal). Axial at *top left*. Three sagittal *slice planes* are shown. Note that layering is visible in all examples, but it is not possible to determinate the exact numbers

**Fig. 5:**
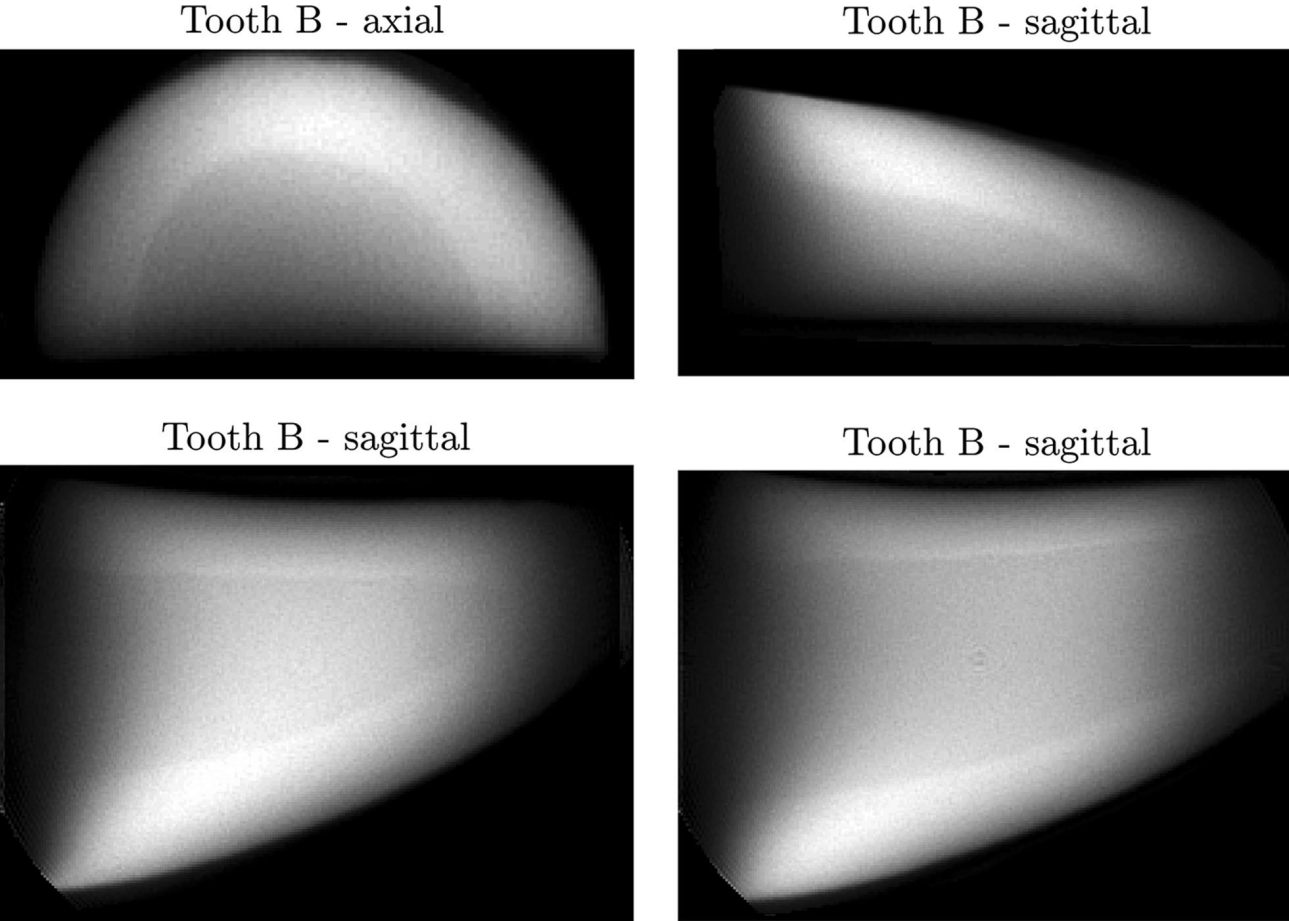
Examples of ZTE images in tooth *B* (see Fig. 1) along various axis (axial and sagittal). Axial at *top left*. Three sagittal *slice planes* are shown. Here, the layered structure is visible as well as the border between marrow and enamel

Teeth were imaged using a 40 mm quadrature coil and a ZTE sequence. For tooth A the scan parameters were: repetition time: 1.92 ms, flip angle: 5°, Bandwidth: 300 kHz, isotropic resolution of 234 µm, 1000 averages were acquired resulting in a total scan time of approximately 21 h. Tooth B was scanned with scan parameters: repetition time 8 ms, flip angle: 10°, Bandwidth: 313 kHz, isotropic resolution of 234 µm, 250 averages were acquired resulting in a total scan time of approximately 22 h. The ZTE sequence does not allow selective excitation and therefore everything within the coil’s field of view contributes to the signal. The edges of the coil field of view are seen in Figs. 4 and 5 as a region where the image fades out. In each image, image contrast has been adjusted to improve visibility of the described features. In all cases contrast was adjusted evenly over the whole image. Remaining image background has been masked for presentation.

### CT method details

Tooth images were imaged on two CT systems using the following scan- and reconstruction parameters.

Siemens Biograph 64: Protocol name: ExtrUHR, 120 kV, 248 eff. mAs, rotation time: 1.0 s, Pitch factor: 0.9, convolution kernel: U70u, slice thickness: 1.0 mm.

Siemens Somatom Definition Flash: Protocol name: Inner Ear UHR: 120 kV, 195 eff. mAs, rotation time: 1.0 s, Pitch factor: 0.8, convolution kernel I: 50 s, slice thickness: 2.0 mm.

## Results

CT scans of whale teeth are shown in Fig. 2 (tooth A and tooth B) and Fig. 3 (tooth B). The CT images from two different clinical CT scanners are shown—the slices are similar, but not exactly from the same plane. The differences between the images are mainly due to different scan settings and reconstruction parameters rather than the CT scanner hardware. The data shown in Fig. 3 was acquired at higher resolution and is consequently more noisy than the data shown in Fig. 2. Higher resolution improves visualization of the fine V-shaped pattern in the tooth, whereas larger structures such as inner holes and different densities of enamel and marrow are clearly visualized at both resolutions. However, only parts of the V-shaped structures can be visualized and the total number of layers cannot be determined from these images. CT scans of the two other teeth gave similar results (data not shown).

The axial ZTE MRI image of tooth A in Fig. 4 (upper left corner) shows concentric ring-like structures. When shown with a sagittal slice orientation (Fig. 4, upper right corner and lower row) V-shaped patterns are seen. Examples of the ZTE scans of tooth B are shown in Fig. 5. Here the contrast delineates the border between cortex and medulla.

**Fig. 6:**
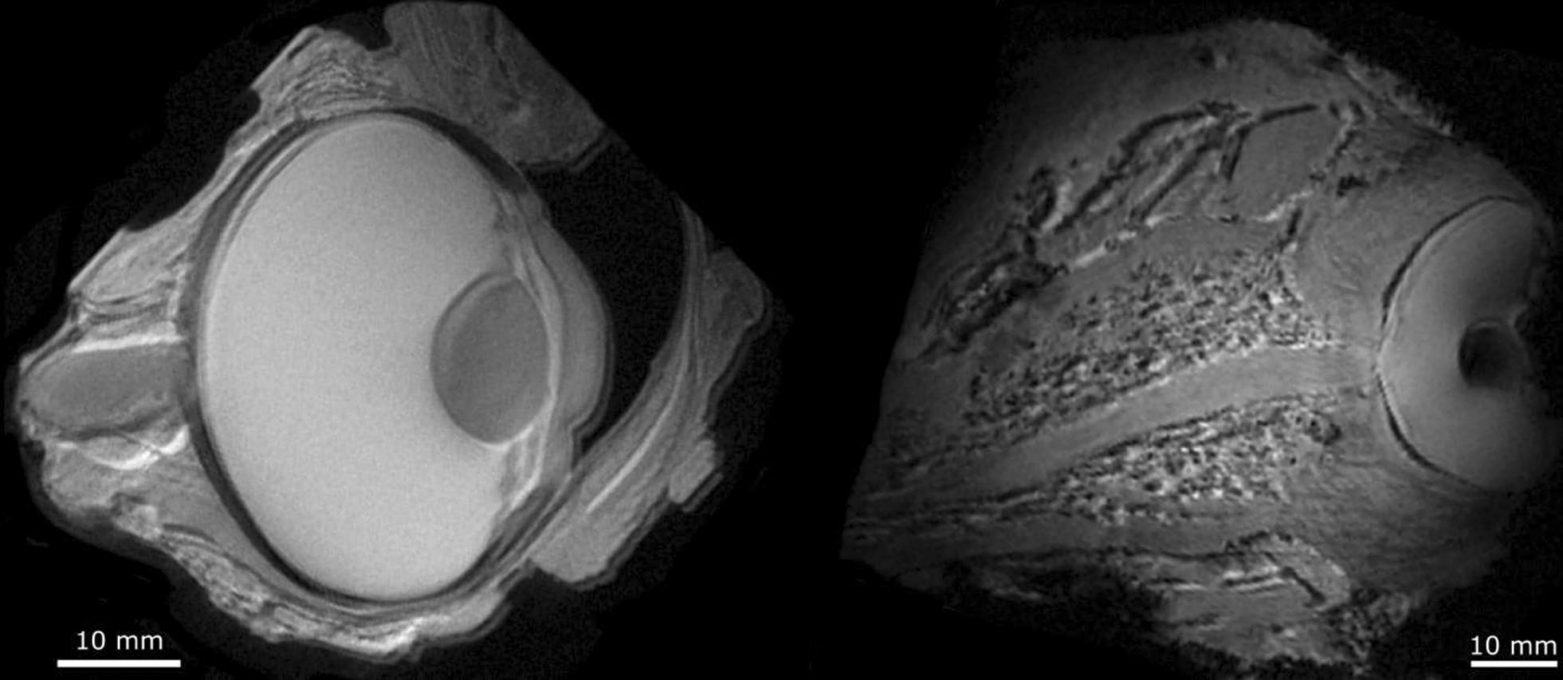
*Left* pig eye scan showing optic nerve, lens, lens suspension, iris and cornea. *Right* whale eye scan showing optic nerve, lens, and lack of internal structures in the eye. Deterioration of the cornea is evident in whale eye

**Fig. 7:**
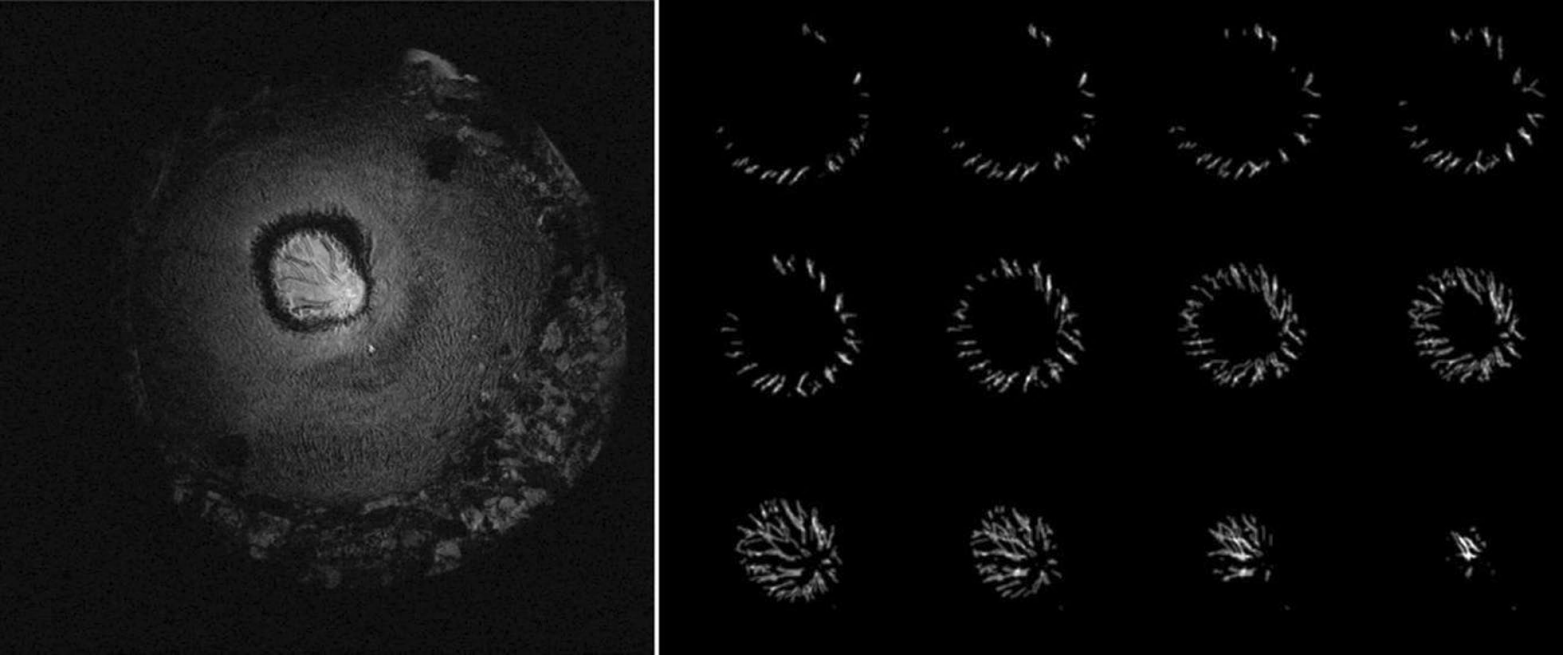
*Left* an example of the visualization of the eye vasculature (elongated dark structures in the *central bright area*) of the whale. *Right* the vascular components isolated by manual segmentation over 12 image planes

Anatomical MRI scans of the pig eye and the significantly larger whale eye is shown in Fig. 6. In contrast to the pig eye, the deterioration was evident in the whale eye. Furthermore, the whale bulbus is seen to be encapsulated by a very large sclera (and remnants of retractor muscles, which were gently tried off-dissected prior to scanning). Neither of these features were prominent in the pig eye. While the pig eye scan very clearly shows optic nerve, lens, lens suspension, iris and cornea, the whale eye scan only shows optic nerve, lens and iris. In fact, the whale eye lacks major internal structures, e.g. the whale lens lack the suspension (ciliary muscles) seen in the pig eye. The whale eye vasculature of the retina was mapped as shown in Fig. 7, which shows an example of a raw image and a montage showing isolated vascular components in each image plane. All blood vessels radiate from the central retina, which is in agreement with position of a central artery and vein, both of which enter the eye together with the optic nerve in the highly vascularized *rete ophthalmica*.

## Discussion

Although adult sperm whales cannot be scanned in commercial scanners, we found that single organ MRI and CT scannings can be suitable tools to gain knowledge of their anatomy. The MRI ZTE sequences and the two different CT scanners showed markedly different performance in elucidating detailed tooth structure. Layering was successfully resolved with MRI in tooth A but was insufficient for age estimation. The concentric appearance of the layers in the axial plane and their V-shaped arrangement in the sagittal plane indicate that the resolved structures are layers in the tooth’s cone-shaped growth zones. In the images of tooth B (sagittal plane), the V-shape of these layers is readily seen along with the distinct border between tooth enamel and marrow (see Fig. 1). In the ZTE scans of tooth B (Fig. 5) this border between enamel and tooth marrow was clearly separated which might be useful for non-destructive studies of variation in enamel thickness in archived samples. This is an underexplored field of marine mammals, though enamel thickness is considered to be crucial for tooth strength in humans and therefore under investigation in dental medicine [29]. Layering, however, could not be resolved in tooth B possibly due to the sample drying out during storage in the museum. In the human teeth study [20] the teeth were stored in water to prevent drying prior to ZTE scans. Future studies might benefit from storing the samples in water for an extended period prior to ZTE imaging. In the case of fresh samples storing the teeth in saline immediately after extraction until imaging might be advantageous. On the CT scans, the border between enamel and marrow was easily seen. The V-shaped layering was resolved but not with sufficient detail to allow age estimation. Higher resolution helps to resolve the layering, but at very high resolutions the noise increasingly becomes a problem. New iterative image reconstruction algorithms are likely to produce images with less noise, but these algorithms were not available for the scanners applied in the present study. In this study, we have not tried high quality scanners, such as micro-CT, since we as well as many other research groups do not have access to such equipment. Therefore, we can not rule out if such scanners would have given images with better contrast and resolution.

The anatomical scans allow qualitative comparison between the whale eye and the terrestic porcine eye. All identified structures (and their size and shapes) were similar to what has already been reported for sperm whale eyes by Bjergager et al. [30] and in agreement with the general cetacean eye described by Mass and Supin [31]. In the pig eye, the scans clearly show the optic nerve, lens, lens suspension, iris and cornea. The whale eye was seen to lack internal structures beyond a lens. The optic nerve is clearly visible, as previously described [30]. The findings of this somewhat superficial comparison of the two eyes are in agreement with what is known about the whale eye anatomy and its use as primarily a light detector without the need for focusing ability [31]. The whale eye is thought to be retractable to withstand the high pressures in the deep sea [30]. The anatomical scans of the whale eye show considerable deterioration of the sample even though the sample was extracted and preserved shortly after the time of death. Even though the pig and whale eyes were treated similarly, degradation was much more pronounced in the latter. This is probably because the large size of the eye, and the whale’s thick sclera, which are both factors that would slow down the diffusion of formalin into the eye and therefore allow more time for sample degradation. It is also possible that the whale died a few hours earlier than the estimated time of death described herein. Consequently, the sample state made it impossible to visualize e.g. the structure of the sclera in this specimen by MRI. However, even in this state the eye vascular network can be visualized as shown in the 2D visualization in Fig. 7. In the present study, we chose to represent the vasculature as a montage of 2D segmentations, but a three dimensional (3D) reconstruction of the vascularization is entirely possible with the acquired data. In more well preserved samples 3D renderings of the nerves, vasculature and connective tissue would be possible with nerve fiber pathways traced by diffusion tensor tractography [32]. In this manner, the MRI would provide a digital dissection of the eye while leaving the sample intact. Modern scanning techniques could offer methods for non-destructive investigation of the musculature allowing such retraction mechanism.

## Conclusions

The use of modern scanning techniques in marine mammal research offers detailed studies of anatomical structures in situ that can increase our knowledge of e.g. anatomical adaptations to an aquatic lifestyle. Even though age could not be estimated based on tooth growing lines, the scanning techniques provided insight into tooth anatomy without the need to cut into the teeth which can be highly desirable for e.g. museum specimens. However, ZTE MRI techniques are continuously being improved and may soon provide sufficient resolution for age estimation. It was possible to get anatomical details of retina vasculation in situ with MRI. However, in soft tissues the state of the sample is the determining factor for successful use of high resolution MRI scanning in marine biology.
